# Mental health of intimate partner violence victims: depression, anxiety, and life satisfaction

**DOI:** 10.3389/fpsyg.2025.1531783

**Published:** 2025-08-11

**Authors:** Maka Lortkipanidze, Nino Javakhishvili, Seth J. Schwartz

**Affiliations:** ^1^School of Arts and Sciences, Ilia State University, Tbilisi, Georgia; ^2^College of Education, University of Texas at Austin, Austin, TX, United States

**Keywords:** depression, anxiety, life satisfaction, intimate partner violence, forms of violence

## Abstract

**Objective:**

Most research on intimate partner violence emphasizes physical and sexual abuse, often overlooking the distinct impacts of different forms of violence on mental health. Psychological abuse is often treated as a single category, overlooking important subtypes like verbal-emotional abuse and controlling behaviors, while economic abuse remains understudied despite its potential impact on well-being. Additionally, life satisfaction—a key indicator of psychological health—is rarely examined alongside depression and anxiety, and the distinction between depressive symptoms and anxiety is often unclear in studies on intimate partner violence. This study addresses these gaps by examining the specific effects of five forms of abuse—physical, sexual, economic, dominance-isolation, and emotional-verbal—on depression, anxiety, and life satisfaction among 293 women survivors of intimate partner violence in Georgia. It also investigates the mediating role of anxiety in the relationship between intimate partner violence and depressive symptoms, thereby focusing on the distinct and unique characteristics of these two mental health outcomes.

**Results:**

The findings revealed that participants commonly experienced at least four forms of intimate partner violence simultaneously, which was associated with adverse mental health outcomes. The impact of intimate partner violence varied by type: physical and sexual abuse were linked to reduced life satisfaction and increased anxiety but were not significant predictors of depressive symptoms. In contrast, dominance-isolation—one form of psychological abuse—predicted depressive symptoms and anxiety but did not affect life satisfaction. Furthermore, anxiety functioned as a mediator in the relationship between physical and sexual intimate partner violence and depressive symptoms, offering insights into the mechanisms underlying the development of depression in abused women.

**Conclusion:**

In conclusion, this study emphasizes the complex relationship between various forms of intimate partner violence and mental health, particularly focusing on the mediating role of anxiety in the development of depressive symptoms. The findings highlight the critical need for the development of comprehensive, culturally sensitive intervention strategies aimed at intimate partner violence victim women, especially those who have endured multiple types of abuse. These interventions should be designed to address the specific mental health challenges associated with diverse forms of interpersonal partner violence, as well as the socio-cultural contexts in which victims reside.

## 1 Introduction

Intimate partner violence (IPV) against women by male partners is a pervasive global issue, cutting across cultural, racial, ethnic, and religious boundaries (Garcia-Moreno et al., 2006). Globally, 27% of ever-married or partnered women aged 15 years and older have experienced physical and/or sexual IPV at least once in their lifetime, with male partners as the perpetrators. The prevalence of IPV is highest in Africa and the South-East Asia region (both at 33%), slightly lower in the Americas (25%), and lowest in high-income countries and Europe (22%; World Health Organization, 2021).

Research demonstrates that intimate partner violence (IPV) predominantly affects women, with 83% of cases involving female victims and male perpetrators (Walby and Towers, 2018). In the United States, 65% of homicides among intimate partners in 2018 involved women as victims, with 96% of these cases perpetrated by male partners (Violence Policy Center, 2018). Tactics such as forced isolation from social networks and controlling behaviors are frequently employed by male abusers (Heise and Garcia-Moreno, 2002; Whitaker, 2015).

The consequences of IPV have increasingly attracted scholarly attention, particularly in the field of violence against women. IPV imposes substantial physical, emotional, and economic burdens on victims, significantly diminishing their quality of life and overall productivity (Karakurt et al., 2014; Fayera and Getachew, 2021). Women subjected to IPV often endure psychological, physical, and economic harm resulting from various forms of abuse. Scholars (Bassuk et al., 2006; Hacıaliefendioğlu et al., 2021) have identified four primary types of IPV—physical, sexual, economic, and psychological abuse—which frequently occur concurrently.

### 1.1 Forms of intimate partner violence

Physical abuse is defined as any intentional aggressive act or threat designed to cause physical harm to another person (Sanderson, 2008). Sexual abuse involves coercive sexual activities initiated by the perpetrator without the consent of the victim, often reflecting dominance and disregard for the woman’s autonomy (Tarzia et al., 2017). Economic abuse entails the perpetrator’s control over family financial resources, restricting access to economic support and financial independence (Davhana-Maselesele et al., 2009).

Definition of psychological abuse, sometimes referred to as emotional abuse, is more complicated and deserves additional clarification. It encompasses both verbal and non-verbal behaviors intended to diminish the victim’s self-esteem and self-worth. Alternative terms include “psychological maltreatment” (Tolman, 1989), “controlling behaviors” (Graham et al., 2006), and “psychological aggression” (Follingstad et al., 2005). Tolman (1992) proposed a continuum of psychological maltreatment, ranging from isolated, less severe acts such as ridicule or emotional insensitivity to more severe forms like extreme intimidation and degradation. Similarly, Follingstad et al. (2005) distinguishes between psychological aggression (e.g., yelling, insults) and coercive tactics (e.g., threats of violence, child custody threats, or forced isolation).

The concept of “coercive control,” also termed “controlling behavior” or “dominance-isolation,” has been described in two ways. It can either represent a method for achieving control within a relationship or function as a subtype of IPV, characterized by severe and gendered patterns of control (Stark, 2007; Walby and Towers, 2018). The distinction between psychological abuse and coercive control is evident in the World Health Organization (2012) definitions, which describe psychological abuse as involving insults, belittling, or intimidation and coercive control as including isolation from social connections and restrictions on financial, educational, or medical access.

The variability in terminology is reflected in psychometric tools for assessing psychological abuse, underscoring the need to differentiate IPV subtypes for their distinct impacts on mental health (Dokkedahl et al., 2019). Moreover, the cultural context influences the effects of specific forms of psychological abuse. For example, in a shame-oriented culture like China, ridicule targeting personal character may have a more profound psychological impact (Bedford and Hwang, 2003). Conversely, in Georgia’s collectivist society, isolation from social networks may be more devastating than verbal abuse, which is often socially normalized (Hofstede, 2020; House et al., 2004).

Given these distinctions, this study separates psychological violence into two components: (1) dominance-isolation, referring to behaviors aimed at isolating the victim from their social network, and (2) emotional-verbal abuse, encompassing verbal attacks, humiliation, and emotional neglect (Tolman, 1995). Consequently, the research examines five forms of IPV: physical, sexual, economic, dominance-isolation, and emotional-verbal abuse.

### 1.2 Intimate partner violence and mental health

Intimate partner violence (IPV) significantly impacts women’s mental health, with consequences including depression, anxiety, post-traumatic stress disorder (PTSD), and reduced life satisfaction (Kilpatrick et al., 2003; Schnurr and Green, 2004; Afifi et al., 2009; Bonomi et al., 2009). Victims frequently report symptoms such as suicidal ideation, persistent sadness, sleep disturbances, and general unhappiness (Karakurt et al., 2014). Studies reveal a 2–3 times higher prevalence of anxiety among IPV survivors compared to non-abused women (Hathaway et al., 2000; Mapayi et al., 2013).

Depression is the most extensively studied mental health outcome related to IPV (Dillon et al., 2013). While many investigations treat IPV as a single construct, findings suggest psychological abuse is a particularly strong predictor of depression. For example, Wong et al. (2011) found psychological abuse to be a significant factor in depression among Chinese women. Additionally, Pico-Alfonso et al. (2006) demonstrated that psychological IPV was as harmful as physical IPV in contributing to depressive symptoms among Spanish women.

Anxiety is another frequently reported outcome of IPV. Studies suggest that the frequency, intensity, and severity of IPV are directly correlated with higher levels of anxiety (Ansara and Hindin, 2011; Savas and Agridag, 2011). Physical, sexual, and psychological violence, often result in both anxiety and depressive symptoms (Wangel et al., 2016; Neilson et al., 2017). While anxiety and depression often co-occur, they are distinguishable constructs. The tripartite model of anxiety and depression (Clark and Watson, 1991) suggests both share negative affect, but anxiety is uniquely associated with physiological hyperarousal, such as somatic tension and dizziness, often triggered by sexual and physical assault (Haskell and Randall, 2019). Accordingly, it is anticipated that victims of physical and sexual IPV are more likely to experience anxiety than depression. Furthermore, research indicates that anxiety may precede and mediate the onset of depression in these cases (Kalin, 2020).

Life satisfaction (LS), a core indicator of psychological well-being (Diener et al., 1985; Diener, 2006), is also negatively impacted by IPV. Abbas and Shah (2017) found that stress associated with IPV significantly reduces LS, while Hui and Constantino (2021) reported that the severity of IPV, such as unwanted sexual experiences, correlates with lower LS. Economic violence also negatively affects LS, often in conjunction with anxiety (Spasovska, 2016) and depression (Postmus et al., 2012). Although there is limited research on the relationship between psychological abuse and LS, studies consistently show a negative association between depression and LS (Stankov, 2013; Moksnes et al., 2016; Vasconcelos et al., 2020). Given that psychological abuse contributes to depressive symptoms and that low life satisfaction is also associated with depression, it can be assumed that women who experience psychological forms of abuse may report lower levels of life satisfaction as a reflection of their diminished well-being.

This evidence emphasizes the need to explore how different forms of IPV uniquely affect mental health outcomes, including depression, anxiety, and life satisfaction, to inform more targeted interventions for victims.

### 1.3 Georgian context

This study was conducted in Georgia, a country that emerged as an independent state following the dissolution of the Soviet Union in 1991. Despite its aspirations to join the European Union, Georgia faces significant challenges, particularly in the areas of poverty and unemployment. According to the Welfare Monitoring Survey (UNICEF, 2018), 5% of the population lives in extreme poverty, while the Caucasus Barometer survey (Caucasus Research Resource Centers, 2019) reports that the primary concerns among respondents are unemployment (48%) and poverty (20%). Due to these economic difficulties, approximately 23% of Georgia’s population has emigrated, seeking opportunities in countries such as Turkey, Russia, various EU member states, and the United States. These challenging economic conditions contribute to an increased risk of violent behavior within the general population (Chitashvili et al., 2010).

The National Survey on Violence Against Women in Georgia (UN Women, 2018) revealed that intimate partner violence (IPV) is widespread, with one in seven women reporting experiences of physical, sexual, and/or emotional violence at the hands of an intimate partner. Specifically, 6% of women reported experiencing physical abuse, 2% reported sexual abuse, and nearly 10% reported economic abuse at some point in their lives.

Culturally, Georgia is considered a patriarchal society with traditional gender roles, and women are significantly underrepresented in decision-making positions across all sectors of public life (Gender Equality Council of the Parliament of Georgia and United Nations Development Programme, 2018). This societal framework may create additional barriers for women experiencing IPV, making it more difficult for them to escape their abusers or seek adequate support (Mestvirishvili et al., 2025).

### 1.4 The present study

While existing research on intimate partner violence (IPV) has predominantly focused on general abuse or one to two types of violence without distinguishing the specific contributions of each form, fewer studies have explored the individual impact of various forms of IPV on mental health outcomes. Most research centers on physical and sexual abuse, while psychological abuse is frequently studied without differentiating between its subtypes, such as verbal-emotional abuse and controlling behaviors that isolate the victim. Additionally, there is a lack of attention to economic abuse, which has been shown to negatively impact the quality of life and may also predict lower life satisfaction (Spasovska, 2016). Moreover, while many studies examine the association of IPV with depression, anxiety, PTSD, and self-esteem, fewer have focused on life satisfaction as a key indicator of psychological well-being.

A notable gap in the literature is the insufficient attention to the differentiation between depressive symptoms and anxiety, even though these constructs can be distinct. The majority of studies emphasize the comorbidity of depression and anxiety among IPV victims, without taking into account the separate effects of each form of IPV on these mental health outcomes.

To address these gaps, this study aims to extend prior research by exploring the effects of five distinct forms of IPV (physical, sexual, economic, dominance-isolation, and emotional-verbal) on mental health variables such as depression, anxiety, and life satisfaction. The primary research question is: do these five forms of IPV have distinct impacts on depressive symptoms, anxiety, and life satisfaction?

### 1.5 Hypotheses

Based on the tripartite model of anxiety and depression, which posits that while anxiety and depression share underlying emotional components, they also have unique characteristics, we developed four hypotheses. According to this model, psychological forms of violence, as well as economic abuse (which resembles psychological violence), are expected to predict both anxiety and depression, while physical and sexual violence, which involve physiological hyper-arousal, are anticipated to primarily predict anxiety, rather than depression.

Additionally, we hypothesize that physical, sexual and economic abuse will negatively impact the victims’ life satisfaction. Given the negative association between depression and life satisfaction, we also expect psychological forms of IPV to adversely predict life satisfaction.

*Hypothesis* 1: All five forms of IPV will positively predict depressive symptoms.

*Hypothesis* 2: All five forms of IPV will positively predict anxiety.

*Hypothesis* 3: All five forms of IPV will negatively predict life satisfaction.

*Hypothesis* 4: The relationship between physical and sexual forms of violence and depressive symptoms will be mediated by anxiety.

These hypotheses aim to address the limitations of prior research by accounting for the distinct contributions of each form of IPV, thereby providing a more nuanced understanding of how different types of violence affect victims’ mental health.

## 2 Methods

### 2.1 Participants

The present quantitative study involved women who were victims of intimate partner violence (IPV). Eligibility for the study required that participants had experienced IPV in an intimate relationship with a husband or another male partner, and that no more than 12 months had passed since the most recent incident of abuse. Participants were recruited from various regions of Georgia, with 20% of the sample coming from women who sought support from governmental and non-governmental agencies, such as crisis centers and shelters. These agencies helped identify eligible participants who were registered in their databases. The remaining 80% of participants were recruited using snowball sampling, where initial participants referred others who met the study’s criteria.

A total of 293 women participated. Of these, 32% resided in the capital city, Tbilisi, while 68% lived in regional towns and villages. The average age of participants was 36 years (±9.75, range: 19–67). The majority of participants (68.6%) lived with their abusive partner, and 40.4% had completed college. While 65% reported having some personal income, only 8% indicated that their income was sufficient to support themselves and their children.

### 2.2 Procedure

Following approval from the university’s ethics board, data collection was carried out by trained^1^ interviewers who were specifically recruited for this study. In some cases, when victims could not leave shelters, interviews were conducted by social workers from the respective agencies. Participants were contacted beforehand, and the purpose of the study was explained to them, with an emphasis on confidentiality. Those who consented to participate were scheduled for interviews in a location away from their home, to ensure their safety from the abuser.

Detailed instructions for filling out the survey were provided to all participants by the interviewers to ensure clarity and ease of understanding. Following this, participants were given paper-based self-report questionnaires to complete individually, which took approximately 30 min. The interviewers remained present during this time, available to assist participants if needed.

### 2.3 Measures

#### 2.3.1 Intimate partner violence

To assess intimate partner violence (IPV), we utilized two primary questionnaires due to the absence of a single tool that comprehensively measures the five distinct forms of IPV targeted in this study. The Psychological Maltreatment of Women Inventory-Short Form Female Version (PMWI; Tolman, 1995) was employed to assess psychological forms of IPV. The PMWI comprises two subscales:

*Dominance-Isolation Subscale* (7 items, *α* = 0.95), measuring behaviors aimed at isolating victims and enforcing subservience, e.g., “My partner interfered in my relationships with other family members.”

*Emotional-Verbal Subscale* (7 items, α = 0.97), assessing verbal abuse and emotional neglect, e.g., “My partner swore at me.”

Additionally, a World Vision study instrument (2013), adapted for the Georgian context, was used to measure physical, economic, and sexual violence. Examples include:

*Physical violence* (7 items): “My partner pushed me, grabbed me, and shook me.”

*Economic violence* (4 items): “I was not allowed to work because my partner did not want me to.”

*Sexual violence* (2 items): “My partner physically forced me to have sex against my will.”

By combining the two instruments, we were able to measure overall IPV (*α* = 0.93) and its distinct forms: emotional-verbal abuse (*α* = 0.90), dominance-isolation (α = 0.89), physical abuse (α = 0.89), economic abuse (α = 0.89), and sexual abuse (α = 0.87).

The items are rated on a 6-point Likert scale (1 = every day, 6 = never). The mean score of each subscale is calculated, resulting in a score for each form of violence ranging from 1 to 6.

#### 2.3.2 Depressive symptoms

To measure depressive symptoms, we used a modified version of the Centers for Epidemiologic Studies Depression Scale (CES-D; Radloff, 1977). The 20-item self-report scale was translated into Georgian and validated (α = 0.89) for Georgian samples (Javakhishvili et al., 2016). Example items include: “I was bothered by things that usually do not bother me” and “I did not feel like eating.” The items are rated on a 4-point Likert scale (0 = rarely or none of the time to 3 = all of the time).

#### 2.3.3 Anxiety

Anxiety symptoms were assessed using the State–Trait Anxiety Inventory (STAI; Spielberger, 1983), specifically the 20-item State Anxiety Scale, translated and validated (α = 0.86) for Georgian samples (Javakhishvili et al., 2016). Sample items include: “I am tense” and “I feel frightened,” with responses scored on a 4-point Likert scale ranging from 1 (not at all) to 4 (very much so).

#### 2.3.4 Life satisfaction

Life satisfaction was measured using the Satisfaction with Life Scale (SWLS; Diener et al., 1985), a 5-item scale that evaluates overall happiness. It has been validated for Georgian samples (α = 0.80; Javakhishvili et al., 2016). Sample items include: “In most ways, my life is close to my ideal” and “The conditions of my life are excellent.” Responses are rated on a 7-point Likert scale (1 = strongly disagree to 7 = strongly agree).

#### 2.3.5 Demographic variables

Demographic data were collected using a separate questionnaire, including questions on participants’ place of residence, age, marital status, educational background, income status, and satisfaction with income.

These measures allowed for a comprehensive assessment of the relationship between various forms of IPV and mental health outcomes, such as depression, anxiety, and life satisfaction, within a Georgian context.

## 3 Results

### 3.1 Descriptive data

All participants in the study had experienced intimate partner violence (IPV) and reported exposure to at least four out of the five forms of IPV studied. A significant portion of participants (69.28%) experienced all five forms of IPV simultaneously. The remaining participants reported combinations of four forms of IPV, with 17.41% experiencing emotional-verbal, dominance-isolation, economic, and physical violence, and 13.31% experiencing emotional-verbal, economic, physical, and sexual violence.

Psychological and economic violence were the most prevalent forms of abuse within the sample. Nearly all participants (99.32%) reported emotional-verbal abuse, 98.63% reported dominance-isolation behaviors, and 93.17% reported economic violence. Physical violence was reported by 81.57% of the participants, and 79.86% experienced sexual violence.

In terms of mental health outcomes, participants significantly elevated levels of depressive symptoms (M = 1.78, SD = 0.59), anxiety (M = 3.12, SD = 0.46), and relatively low life satisfaction (M = 2.09, SD = 1.03). These findings suggest a clear negative impact of IPV on both emotional well-being and overall quality of life.

### 3.2 Correlations

Table 1 presents the correlations between the frequency of different forms of intimate partner violence (IPV) and various mental health outcome variables, including depression, anxiety, and life satisfaction. The results showed that all five forms of IPV—emotional-verbal abuse, dominance-isolation, economic abuse, physical abuse, and sexual abuse—were significantly and positively correlated with both anxiety and depression, and negatively correlated with life satisfaction. These correlations were all statistically significant and in the expected direction, with the strength of the relationships varying from weak to moderate.

**Table 1:** Correlations between IPV and mental health variables.

Forms of IPV	Depression	Anxiety	Life satisfaction
Emotional-Verbal	0.13*	0.38**	−0.43**
Dominance-Isolation	0.25**	0.27**	−0.28**
Economic	0.26**	0.32**	−0.29**
Physical	0.20**	0.41**	−0.31**
Sexual	0.17**	0.24**	−0.18**

*p < 0.05. **p < 0.01.

### 3.3 Hypothesis testing

To evaluate our first three hypotheses and identify the predictors of mental health outcomes (depressive symptoms, anxiety, and life satisfaction), hierarchical regression analyses were conducted. In the first model, demographic variables (place of residence, age, marital status, education, income satisfaction) were entered, followed by IPV forms (physical, sexual, economic, emotional-verbal, and dominance-isolation) in the second model. The results of the final model are presented in Table 2.

**Table 2:** Standardized regression coefficients for depressive symptoms, anxiety and life satisfaction.

Model		Depressive symptoms		Anxiety		Life satisfaction	
		*β*	*t*	*β*	*t*	*β*	*t*
1	Place of Residence	−0.13	−1.78	−0.48**	−6.80	0.14	1.72
	Education Level	−0.20*	−2.42	−0.01	−0.17	0.25**	3.57
	Satisfaction with Income	−0.24**	−3.35	−0.24**	−3.82	0.19*	2.84
2	Place of Residence	−0.06	−0.95	−0.44**	−6.09	0.03	0.41
	Education Level	−0.20*	−2.60	−0.01	−0.20	0.15*	2.39
	Satisfaction with Income	−0.26**	−3.74	−0.24**	−3.90	0.20**	3.10
	Economic Abuse	0.08	1.01	0.03	0.20	−0.08	−0.99
	Emotional-Verbal Abuse	0.00	0.02	0.03	0.39	−0.02	−0.25
	Dominance-Isolation	0.32**	3.91	0.19*	2.84	−0.09	−1.26
	Physical Abuse	0.05	0.62	0.19*	2.46	−0.26*	−2.80
	Sexual Abuse	0.05	0.63	0.18*	2.72	−0.19*	−2.40

*p < 0.05. **p < 0.01.

For depressive symptoms, the overall regression model was significant, *F*(5,193) = 7.59, *p* < 0.01, R^2^ = 0.30. The demographic variables, specifically education level and satisfaction with personal income, explained 16% of the variability—F(5,193) = 7.59, *p* < 0.01, R^2^ = 0.16, while the dominance-isolation accounted for an additional 14% of the variance in depressive symptoms scores, F(5,193) = 7.59, *p* < 0.01, R^2^ = 0.14.

Regarding anxiety, the regression model was also significant, F(5,193) = 2.55, *p* < 0.05, R^2^ = 0.42. Place of residence and satisfaction with personal income together explained 38% of the variability in anxiety scores—F(5,193) = 2.55, *p* < 0.05, R^2^ = 0.38, while IPV forms, including dominance-isolation, physical, and sexual abuse, contributed an additional 4% to the variance, F(5,193) = 2.55, *p* < 0.05, R^2^ = 0.04.

The regression model for life satisfaction was significant as well, F(5,193) = 8.94, *p* < 0.01, R^2^ = 0.37. Education and satisfaction with personal income accounted for 22% of the variability in life satisfaction—F(5,193) = 8.94, *p* < 0.01, R^2^ = 0.22, with physical and sexual abuse explaining an additional 15% of the variance, F(5,193) = 8.94, *p* < 0.01, R^2^ = 0.15.

Thus, Hypothesis (1) was partially supported: only dominance-isolation emerged as a significant positive predictor of depressive symptoms, while none of the other forms of abuse independently predicted the outcome.

Hypothesis (2) was likewise partially supported: economic and emotional-verbal abuse did not independently predict anxiety, whereas only dominance-isolation, physical, and sexual abuse emerged as significant predictors.

Hypothesis (3) also received partial support: physical and sexual abuse significantly predicted lower life satisfaction, whereas none of the psychological forms of abuse nor economic abuse independently predicted life satisfaction among the victims.

As indicated in the table, demographic variables also proved to be significant predictors of the outcomes. Satisfaction with personal income was found to predict all three mental health variables (depressive symptoms, anxiety, and life satisfaction), while education level was associated with depressive symptoms and life satisfaction. Additionally, place of residence was negatively associated with anxiety. It is important to note that for depressive symptoms and anxiety, demographic variables accounted for a larger proportion of the variance than the primary IPV forms. Conversely, life satisfaction was more strongly predicted by the different forms of IPV than by the demographic factors.

**Figure 1:**
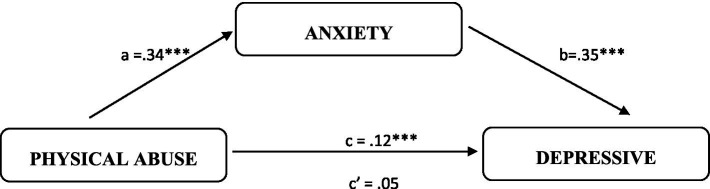
Summary of the direct and indirect effects of physical abuse on depressive symptoms through anxiety. ***p < 0.01.

**Figure 2:**
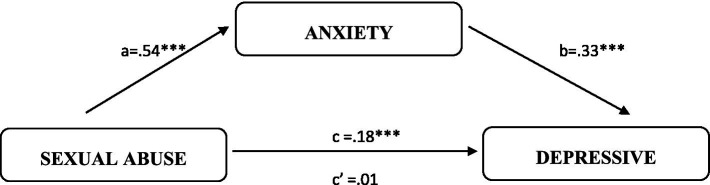
Summary of the direct and indirect effects of sexual abuse on depressive symptoms through anxiety. ***p < 0.01.

To test the fourth hypothesis, we conducted a mediation analysis using the PROCESS macro (version 3.5, Hayes, 2017). The results are illustrated in Figures 1, 2.

As shown in Figure 1, physical abuse was found to significantly predict increased anxiety [*β* = 0.34, t(291) = 7.26, *p* < 0.01] among IPV victims. In turn, anxiety predicted an increase in depressive symptoms [*β* = 0.35, t(290) = 5.59, *p* < 0.01]. The bootstrap confidence interval for the indirect effect of physical abuse on depressive symptoms via anxiety (*β* = 0.12) did not include zero (95% CI = 0.06 to 0.18), confirming a significant mediation effect. The direct effect of physical abuse on depressive symptoms was not significant [*β* = 0.05, t(291) = 0.89, *p* > 0.05]. The overall mediation model was significant, with a total effect of 0.17 [t(291) = 3.18, *p* < 0.01].

Figure 2 illustrates the mediation effect of anxiety between sexual abuse and depressive symptoms. Sexual abuse significantly predicted increased anxiety [*β* = 0.54, t(291) = 3.84, *p* < 0.01], and anxiety, in turn, positively predicted depressive symptoms [*β* = 0.33, t(290) = 5.69, *p* < 0.01]. The bootstrap confidence interval for the indirect effect of sexual abuse on depressive symptoms via anxiety (*β* = 0.18) was entirely above zero (95% CI = 0.06 to 0.33), indicating a significant mediation effect. The direct effect of sexual abuse on depressive symptoms was not significant [*β* = 0.01, t(290) = 0.08, *p* > 0.05]. The overall mediation model was significant, with a total effect of 0.19 [t(291) = 1.99, *p* < 0.01].

Hypothesis (4) was supported, with full mediation observed in both cases. Specifically, anxiety fully mediated the relationship between physical abuse and depressive symptoms, as well as the relationship between sexual abuse and depressive symptoms. This indicates that while physical and sexual abuse directly affect anxiety, anxiety itself plays a central role in contributing to depressive symptoms.

## 4 Discussion

The present study sought to examine the distinct effects of various forms of intimate partner violence (IPV)—physical, sexual, economic, dominance-isolation, and emotional-verbal abuse—on mental health outcomes (depression, anxiety, and life satisfaction), as well as the mediating role of anxiety in the relationship between IPV and depressive symptoms.

Consistent with our hypotheses, the findings indicate that not all forms of IPV have the same impact on mental health, and different types of violence are associated with distinct mental health outcomes. Among the five forms of IPV examined, dominance-isolation (a form of psychological abuse) as well as physical and sexual abuse emerged as significant predictors of poor mental health outcomes for Georgian women. Specifically, physical and sexual abuse were associated with lower life satisfaction and higher anxiety but did not independently predict depression. Conversely, dominance-isolation predicted both depression and anxiety but did not independently affect life satisfaction. These findings may be attributed to our simultaneous analysis of the five forms of IPV, a model that, to the best of our knowledge, has not been previously studied in relation to three different mental health outcomes. Our descriptive data also show that IPV victims commonly experience multiple forms of abuse alongside mental health challenges, highlighting the complexity of the lived experiences of these individuals.

In contrast to findings from other regions (Afifi et al., 2009; Bonomi et al., 2009; Karakurt et al., 2014), economic and emotional-verbal abuse did not independently predict any of the mental health outcomes in this study. This divergence may be attributed to the cultural context in Georgia, where emotional-verbal abuse is often seen as a normative aspect of marital relations, particularly in a patriarchal society (UN Joint Programme for Gender Equality, 2013). In Georgia, verbal abuse is not always recognized as psychological violence but rather as an acceptable form of behavior within the framework of gender roles. The relative deprivation theory further suggests that economic abuse may have less of an impact in a context of widespread societal hardship, where the relative disadvantage is less pronounced (Walker and Pettigrew, 1984). Furthermore, economic violence is not always clearly recognized by the victim women, as it is frequently masked by other forms of abuse, such as controlling behaviors that lead to dominance-isolation (Mestvirishvili et al., 2025).

A key finding of the study is that the relationship between IPV and mental health outcomes may not always be direct; rather, the link can be mediated by other mental health variables, as supported by the mediation analysis. Contrary to expectations, no direct effect was found between physical and sexual abuse and depressive symptoms. Instead, the impact of physical and sexual abuse on depressive symptoms was fully mediated by anxiety. This result aligns with theories of comorbidity between anxiety and depression, where anxiety is seen as a precursor to depression, particularly in women (Chorpita and Daleiden, 2002; Kalin, 2020).

These findings support the tripartite model, which posits that depression and anxiety share both common and distinct features (Hunt et al., 2002). Anxiety, linked to physiological hyper-arousal, appears to result from physical and sexual abuse, while psychological abuse leads to comorbid depression-anxiety, a finding consistent with prior research (Abdelhai and Mosleh, 2015; Pickover et al., 2017). In the case of psychological abuse, humiliation and forced submission often erode self-esteem, contributing to the development of depression in women (Mechanic et al., 2008; Dokkedahl et al., 2019).

In terms of life satisfaction, physical and sexual violence were associated with decreased life satisfaction, aligning with previous studies (Hui and Constantino, 2021). This may be explained by the fear and insecurity induced by violence, which negatively affects overall well-being (Poutiainen and Holma, 2013). Among the psychological forms of IPV, dominance-isolation emerged as a significant predictor of mental health, which may be reflective of Georgia’s collectivist culture, where familial and social connections are integral (Hofstede, 2020).

Additionally, demographic factors such as place of residence, education level, and satisfaction with personal income were significant predictors of mental health outcomes in our sample. Specifically, women dissatisfied with their personal income showed poor mental health across all outcomes, reflecting the socio-economic challenges faced in Georgia. Women with low educational attainment reported lower life satisfaction and higher depressive symptoms, while anxiety was more prevalent among women in rural areas. Interestingly, living in rural areas without sufficient personal income had a stronger impact on anxiety than IPV itself, potentially due to the pervasive poverty and limited employment opportunities in rural Georgia (Asian Development Bank, 2022).

Finally, the mental health outcomes of women who remained with their abusive partners did not differ significantly from those who had separated from the perpetrator. This suggests that the psychological effects of IPV persist regardless of relationship status, with some victims reporting enduring psychological problems even years after separation.

In conclusion, this study highlights the complex relationship between different forms of IPV and mental health, with a particular emphasis on the mediating role of anxiety in the development of depressive symptoms. These findings underscore the importance of addressing both the direct and indirect psychological impacts of IPV, especially in socio-cultural contexts like Georgia, where traditional gender norms may influence the recognition and response to various forms of abuse.

## 5 Implications

The findings of this study highlight several key implications for the development of targeted intervention programs for women experiencing intimate partner violence (IPV). These implications are particularly important for stakeholders such as governmental bodies, non-governmental organizations (NGOs), healthcare providers, and mental health professionals involved in supporting victims of IPV.

First, it is critical for service providers to recognize that victims of IPV often endure multiple forms of abuse concurrently, and that these forms can have distinct and varied impacts on mental health. The results from this study indicate that women suffering from IPV are frequently subjected to at least four different forms of violence, each contributing differently to their mental health outcomes. As such, professionals working with IPV victims must be well-informed about the specific types of abuse that individuals experience in order to provide tailored mental health support. For example, those working with women who have experienced physical or sexual abuse should anticipate elevated levels of anxiety, which may act as a precursor to depressive symptoms. Conversely, for women who have suffered from dominance-isolation, a form of psychological abuse, there is a co-occurrence of both anxiety and depression, which demands a different approach to intervention.

Given the significant role that anxiety plays in mediating the relationship between IPV and depressive symptoms, particularly for women who have experienced physical or sexual abuse, it is essential that intervention strategies prioritize the reduction of anxiety. This could involve providing psychological therapies focused on anxiety management, such as cognitive behavioral therapy (CBT), which has been shown to be effective in treating anxiety disorders (Hofmann et al., 2012). Early intervention focused on anxiety may, in turn, help prevent the onset of depression, which can exacerbate the negative consequences of IPV on mental health. Therefore, treatment programs for IPV victims should emphasize the identification and management of anxiety symptoms, which could significantly improve the mental health outcomes for survivors of physical and sexual forms of abuse.

Furthermore, the study found that women residing in rural areas were disproportionately affected by IPV, as they face not only the psychological and physical consequences of abuse but also socio-economic challenges, such as limited access to healthcare, financial instability, and social isolation. Rural women were found to have higher levels of anxiety compared to their urban counterparts, suggesting that economic hardships and lack of resources may further exacerbate their mental health difficulties (Logan et al., 2003). This finding underscores the need for targeted interventions for rural women that address both the mental health consequences of IPV and the economic factors that contribute to their anxiety. Programs that provide economic support, increase access to healthcare, and create safe spaces for social support could help alleviate the additional stressors faced by these women.

Moreover, the study highlighted the significant negative impact of physical and sexual abuse on life satisfaction. Victims of these forms of IPV reported low levels of life satisfaction, indicating that they may be at an elevated risk for experiencing a diminished quality of life. This finding has important implications for the design of support services, particularly in terms of providing safe and sustainable living conditions for women attempting to escape abusive relationships. It is crucial for governmental and non-governmental agencies to enhance their efforts to offer shelter services, with particular attention to extending the length of stay for women in these facilities. Long-term housing support could enable victims of physical and sexual forms of violence to regain their independence and improve their overall well-being (Logan et al., 2003).

Given the critical role of life satisfaction in the recovery process, it is also essential for intervention programs to focus on helping victims rebuild their lives by offering practical support in securing safe housing, employment, and financial independence. Such interventions can empower women to leave abusive relationships and start afresh in a safe environment, reducing the psychological burden of IPV and improving their quality of life (Coker et al., 2002). Additionally, offering therapeutic interventions that focus on rebuilding self-esteem and providing emotional support is crucial in helping victims regain a sense of personal control and well-being.

In conclusion, the findings from this study emphasize the importance of implementing comprehensive and culturally sensitive intervention programs for IPV victims, particularly those who have experienced multiple forms of abuse. These programs should address the specific mental health needs of survivors, with a focus on anxiety reduction and improving life satisfaction. Furthermore, interventions must consider the socio-economic context in which IPV victims live, particularly for women in rural areas, and ensure that resources are available to support them in escaping abusive situations. By tailoring interventions to the unique needs of IPV victims, stakeholders can more effectively mitigate the long-term psychological and emotional consequences of abuse.

## 6 Conclusion and limitations

The results of this study must be interpreted with consideration of several key limitations. First, the findings can only be generalized to female victims of intimate partner violence (IPV) who share certain demographic characteristics, such as lower socioeconomic status and limited educational attainment. This is consistent with previous research, which has highlighted the difficulty of recruiting participants from this population, as many victims feel shame or fear social stigma associated with disclosing their experiences of abuse (Javakhishvili and Jibladze, 2018). This challenge is particularly pronounced in Georgia, where cultural norms regard domestic violence as a private family matter, thus discouraging open discussion and reporting of such abuse. Consequently, our sample may not fully represent the broader population of IPV victims, especially those from different socio-economic backgrounds or cultural contexts. At the same time, the study’s focus on a non-WEIRD context provides a meaningful contribution by highlighting IPV experiences in underrepresented cultural settings.

Another limitation is the use of a cross-sectional design, which precludes the ability to draw definitive causal conclusions. Although the study identifies significant associations between various forms of IPV and mental health outcomes, the design does not allow for an examination of the temporal relationship between these variables. Longitudinal research would be necessary to better understand the causality and long-term effects of IPV on mental health.

Despite these limitations, the study’s findings offer valuable insights into the mental health consequences of IPV. Notably, anxiety emerged as the most sensitive mental health indicator, followed by life satisfaction, while depression appeared to be less responsive to IPV. These results suggest that anxiety may serve as an early marker of psychological distress among IPV victims. Additionally, not all forms of IPV were found to contribute equally to mental health problems. Economic violence, although prevalent, did not uniquely predict mental health outcomes in this sample, which aligns with previous findings in other cultural contexts (Afifi et al., 2009; Antai et al., 2014). Furthermore, the study highlights the need to break down psychological violence into more specific components, as only dominance-isolation was significantly associated with poor mental health, in contrast to other forms of abuse.

The findings also underscore the importance of considering contextual factors when examining the relationship between IPV and mental health. While our results differ somewhat from those reported in studies from other countries, they point to the significant role that cultural, economic, and social contexts play in shaping the impact of IPV on victims. These results suggest that interventions designed to address IPV and its mental health consequences should be tailored to the specific cultural and socio-economic context of the affected population.

In conclusion, while the study’s limitations must be acknowledged, the findings contribute to a deeper understanding of the complex relationship between IPV and mental health. The results suggest important avenues for future research, particularly in exploring the role of contextual factors and the specific pathways through which IPV impacts mental health. We hope that our study will stimulate further research in this area, ultimately leading to more effective and context-sensitive interventions for IPV victims.

## Data Availability

The original contributions presented in the study are included in the article/supplementary material, further inquiries can be directed to the corresponding author.

## Footnotes

^1^A two-day training on domestic violence, including its causes and consequences, was provided to all interviewers, including agency representatives, by one of the authors of this article.
